# Association between cardiovascular health and perceived quality of life in ethnically diverse adults: insights from the Community of Mine study using the American Heart Association’s Life’s Simple 7

**DOI:** 10.1007/s11136-024-03853-3

**Published:** 2024-12-18

**Authors:** E. J. Ambeba, D. D. Sears, T. Benmarhnia, L. Natarajan, S. Zamora, S. Alismail, C. P. Tribby, M. M. Jankowska

**Affiliations:** 1 Herbert Wertheim School of Public Health & Human Longevity Science, University of California, La Jolla, San Diego, CA 92093 USA; 2 College of Health Solutions, Arizona State University, 550 N. 3rd St., Phoenix, AZ 85004 USA; 3 Department of Medicine, UC San Diego, La Jolla, CA 92093 USA; 4 Department of Family Medicine, UC San Diego, La Jolla, CA 92093 USA; 5 Scripps Institution of Oceanography, UCSD, 9500 Gilman Drive, La Jolla, CA 92093 USA; 6 Population Sciences, Beckman Research Institute, City of Hope, 1500 E Duarte Rd, Duarte, CA 91010 USA

**Keywords:** Subjective quality of life, Lifestyle recommendations, Risk factors, Atherosclerosis, Coronary heart disease

## Abstract

**Purpose:**

The association between cardiovascular health (CVH) with perceived quality of life (PQoL) and variations by sex and Hispanic ethnicity is not well understood.

**Methods:**

This study included 583 participants (42% Hispanic, 56% female, mean age 59 years). Linear regression modeled the covariate-adjusted associations between CVH, using the combined 7 components of Life’s Simple 7 (LS7; ideal and intermediate, compared to poor), and PQoL (total and physical, social, and cognitive health domains). For individual LS7 components, we assessed effect modification by sex and Hispanic ethnicity.

**Results:**

Compared to individuals with poor CVH, those with intermediate (β [95% CI] = 0.22 [0.09, 0.35]) and ideal (β [95% CI] = 0.22 [0.08, 0.36]) CVH had higher overall PQoL. This effect was dominated by the physical PQoL domain. Of LS7 components, ideal body mass index (BMI) (β [95% CI] = 0.17 [0.03, 0.31]) and physical activity (β [95% CI] = 0.26 [0.12, 0.40]) were associated with overall PQoL. Ideal diet (β [95% CI] = 0.32 [0.08, 0.56]) and fasting plasma glucose (β [95% CI] = 0.32 [0.06, 0.58]) were associated with the physical PQoL domain. A higher PQoL score was associated with intermediate BMI in women, and physical PQoL was associated with smoking for women. A BMI*Hispanic interaction resulted in larger associations between intermediate/ideal BMI and physical PQoL in non-Hispanics.

**Conclusion:**

Ideal or intermediate CVH health factors and health behaviors were associated with higher PQoL. Sex and ethnicity differences suggest that perceived quality of life is associated with BMI for women and non-Hispanics.

**Supplementary Information:**

The online version contains supplementary material available at 10.1007/s11136-024-03853-3.

## Introduction

Maintaining a favorable cardiovascular risk profile is essential for preventing morbidity and mortality from cardiovascular disease (CVD) [1]. The American Heart Association’s Life’s Simple 7 (LS7) defines ideal cardiovascular health (CVH) as meeting goals in three health behaviors (dietary pattern, physical activity, and smoking cessation) and four health factors (body mass index (BMI), total cholesterol, fasting plasma glucose (FPG), and blood pressure (BP)) [2]. Studies have shown that the combination of health behaviors and health factors is strongly and inversely associated with all-cause mortality, CVD morbidity and mortality, and heart failure [3, 4]. Specifically, LS7 has been well validated and is highly predictive of incident major cardiovascular events [5, 6].

Unfavorable health behaviors and factors linked to an increased risk of CVD have generally been associated with reduced quality of life (QoL) [7–12]. Few studies have investigated the association between QoL and ideal CVH as defined through LS7. Two studies examined ideal CVH in relation to objective measures of QoL (e.g., loss of healthy days due to physical or mental illness) [13, 14]. Both studies found that ideal CVH was associated with a reduced prevalence of physically and mentally unhealthy days. However, the association between ideal CVH and PQoL remains poorly understood. There is some evidence to suggest that sex [15] and racial/ethnic differences [16–18] influence the association between individual LS7 components, particularly health behaviors such as physical activity and BMI, and QoL.

This study examined the association between CVH and PQoL, with specific research questions: (1) Is ideal CVH, as defined by LS7, associated with better overall PQoL, and PQoL in the physical, cognitive, and social domains? (2) Are ideal levels of LS7 components associated with better overall and domain-specific PQoL? (3) Do sex and Hispanic ethnicity modify the associations between LS7 components and PQoL? We hypothesized that participants with ideal overall CVH and LS7 components will have higher overall and domain-specific PQoL than those with poor CVH. We further hypothesized that the associations for health behavior LS7 components (diet, physical activity, and smoking) will differ between men and women. Finally, we hypothesized that the association between BMI and PQoL will be modified by Hispanic ethnicity [19].

## Methods

### Study design and participants

The Community of Mine study was a cross-sectional observational study conducted in San Diego County, California with the primary objective of observing associations between built environment exposure and biomarker outcomes in an ethnically diverse sample of adults. The data that support the findings of this study are available from the corresponding author upon reasonable request. The full study protocol is published elsewhere [20]. Briefly, 602 adult participants (ages 35 to 80) were selected from a stratified random sample from urban and suburban census block groups to maximize variability in built environment walkability (sample enrollment workflow in Supplement Fig. 1). Inclusion criteria were participants must have lived in a census block group selected for the study for at least 6 months, be able to walk without human assistance, be able to travel to a study site, have a phone, be able to read and write fluently in English or Spanish, be able to give informed consent and comply with the protocol, and be willing and able to complete all assessments [20]. Exclusion criteria were being pregnant or nursing, having a mental state that would preclude complete understanding of the protocol or compliance, having a medical condition that would affect PA or diet, or having a medical condition known to increase inflammation biomarkers [20].

Data was collected between 2014 and 2017. Signed informed consent was obtained from all participants who enrolled in the study. Upon receipt of the signed consent form, accelerometers were mailed to participants along with questionnaires. After wearing the devices for 1 week, participants attended a weekday clinic visit, during which anthropometric measures, vitals, a 24-h food recall, medication being taken, and a fasting blood sample was collected. After the visit, participants wore the study devices for an additional week and one additional food recall was collected by phone interview. The study was approved by the University of California, San Diego (UCSD) Institutional Review Board (Protocol #140510).

### Measurement of LS7 components

#### Smoking status

Cigarette smoking status was determined from a question on self-reported smoking history: current smoker, former smoker, or never smoker. The AHA’s original definition considers individuals who never smoked or were former smokers who quit > 12 months prior as ideal, former smokers who quit < 12 months prior as intermediate, and those who were current smokers as poor [2]. In our study, only six former smokers had quit ≤ 12 months prior; therefore, we considered ideal to be limited to those who had never smoked, and intermediate to include those who were former smokers, regardless of their quit year (Table 1).

**Table 1:** Definitions of ideal, intermediate, and poor for each component of the American Heart Association’s Life’s Simple 7

Measures	Level of CVH for each component		
	Ideal	Intermediate	Poor
Smoking status	Never smoker	Former smoker	Current smoker
Healthy diet	4 to 5 components	2 to 3 components	0 to 1 component
Physical activity	≥ 21.4 min/day	10 to < 21.4 min/day	< 10 min/day
Body mass index	< 25 kg/m^2^	25 to < 30 kg/m^2^	≥ 30 kg/m^2^
Total fasting cholesterol	< 200 mg/dL (not treated)	200 to 239 mg/dL or treated to goal	≥ 240 mg/dL
Blood pressure*	SBP < 120 mm Hg and DBP < 80 mm Hg (not treated)	SBP 120 to 139 mm Hg or DBP 80 to 89 mm Hg or treated to goal	SBP ≥ 140 mm Hg or DBP ≥ 90 mm Hg
Fasting plasma glucose	< 100 mg/dL (not treated)	100 to 125 mg/dL or treated to goal	≥ 126 mg/dL

*SBP, systolic blood pressure; DBP, diastolic blood pressure

#### Body mass index

Height and weight, measured without shoes, were recorded twice using a stadiometer and a bariatric digital scale. BMI was calculated as average weight (kg)/height (m^2^). BMI was categorized: ideal, < 25 kg/m^2^; intermediate, 25 to < 30 kg/m^2^; or poor, ≥ 30 kg/m^2^ (Table 1).

#### Physical activity

We used objectively measured PA to enhance reliability and reduce error and misclassification bias of self-report [21]. We measured PA using an ActiGraph GT3X + accelerometer (ActiGraph, LLC; Pensacola, FL) worn on the hip during waking hours. The raw accelerometer data (collected at 30 Hz on 3 axes) were processed with the low frequency extension to the minute level using Actilife 6 software (ActiGraph, LLC; Pensacola, FL). Within this software, non-wear time was identified and excluded using the validated Choi algorithm, defined as 90 consecutive minutes of zero counts with a two-minute tolerance and a 30-min window to detect artificial movement [22]. Participants had valid data if they had at least four adherent days with at least 10 h of wear time per day. Moderate to Vigorous PA was determined using accelerometer counts per minute (cpm) cut-offs (MVPA; ≥ 1,952 cpm) [23]. Participants were classified as having ideal PA if they averaged at least 21.4 min of MVPA per day (approximately 150 min per week) meeting the recommended amount of PA per AHA guidelines [24]. Participants were classified as intermediate for 10 to < 21.4 min/day or poor for < 10 min/day (Table 1).

#### Healthy diet

Dietary measures were extracted from two 24-h, multiple-pass food recalls (one collected on a weekday and one on a weekend day) using the Nutrition Data System for Research (NDSR) versions 2015 and 2016 [25]. Participants with at least two reliable recalls were included. Reliability was determined by a research assistant (and quality checked by the NDSR master trainer). A food recall was categorized as unreliable, for example if the participant stated they had difficulty recalling food ate or amounts; if the participant did not want to thoroughly complete the food recall interview; or if the participant gave food portion amounts that seemed unlikely. A healthy diet was defined as meeting the recommended intake levels for five components: (1) ≥ 4.5 servings of fruits and vegetables per day; (2) ≥ 7 oz of fish per week; (3) ≥ 3 oz of fiber‐rich whole grains per day (≥ 1.1 g of dietary fiber/10 g of carbohydrate per day); (4) < 1500 mg of sodium per day; and (5) ≤ 36 oz of sugar‐sweetened beverages (SSB) per week. A score of 0 indicated that the participant did not meet any recommended levels, while a score of 5 indicated that they met the recommended levels for all five components. Fish and SSB intakes were modified from the original AHA healthy diet measure (2) to reflect daily amounts of each per day, based on the design of the Community of Mine study [20]. Participants’ healthy diet category was classified as ideal for meeting 4 or 5 components; intermediate for meeting 2 or 3 components; or poor for meeting 0 or 1 components (Table 1).

#### Blood pressure

BP measures were obtained by clinic staff during the clinic visit using an automated BP monitor (GE Dinamap Carescape V100 or Accutorr 7). BP was measured three times with a one-minute rest between readings, and a fourth measure was taken if two of the three previous readings differed by greater than five mm Hg. BP was measured prior to any other visit activity and after the participant was seated quietly for 10 min. Participants’ blood pressure category was classified as ideal for systolic blood pressure (SBP) < 120 mm Hg and diastolic blood pressure (DBP) < 80 mm Hg (not treated with medication); intermediate for SBP 120 to 139 mm Hg or DBP 80 to 89 mm Hg or treated with medication to goal; or poor for SBP ≥ 140 mm Hg or DBP ≥ 90 mm Hg (Table 1).

#### Fasting plasma glucose and total cholesterol

Participants fasted for 12 h before blood draws during their clinic visit. Blood samples were held on ice for 60 min before centrifugation and aliquoting. Glucose concentration was measured in plasma isolated from blood drawn into EDTA tubes, which were centrifuged at 4 °C, then aliquoted, and stored at −80 °C. Glucose concentration (mg/dL) was measured in all samples at the end of the Community of Mine study using a standard glucose oxidase method (YSI 2900 Bioanalyzer), with intra-assay coefficient of variance of 3.2%. Total cholesterol concentration was measured on the day of clinic visit from plasma collected in plasma separator tubes at the UCSD Center for Advanced Laboratory Methods. Participants’ fasting plasma glucose category was classified as ideal for < 100 mg/dL (not treated); intermediate for 100 to 125 mg/dL or treated with medication to goal; or poor for ≥ 126 mg/dL (Table 1). Participants’ total cholesterol category was classified as ideal for < 200 mg/dL (not treated); intermediate for 200 to 239 mg/dL or treated with medication to goal; or poor for ≥ 240 mg/dL (Table 1).

#### LS7 composite measure

Each LS7 component was assigned 0 points for poor, 1 point for intermediate, and 2 points for ideal [26], as described in Table 1. For fasting cholesterol, blood pressure, and fasting plasma glucose the study team coded medications being taken by participants for treatment of these conditions. Three measures of LS7 were developed: (1) the composite LS7 score (overall CVH) was computed by summing the seven component scores, resulting in a possible range of 0 to 14, (2) tertiles of the composite LS7 score were used to classify participants as having poor (score of 0–7), intermediate (score of 8–9), or ideal (score of 10–14) CVH, and (3) the total number of ideal LS7 components was computed per participant as an ordinal variable.

### Perceived quality of life

PQoL was assessed using the Perceived Quality of Life scale, a subjective assessment of life circumstances [27], originally developed as a self-report instrument with 12 items to evaluate outcomes of intensive care treatment [28]. Patrick et al. later added eight additional items related to satisfaction with functional status, including the item “how happy are you?”, which was used to examine convergent validity within the instrument [27]. For the current study, a modified version of the original and Patrick et al.’s scale was utilized, consisting of 12 items. Responses for each item were measured on a 5-point Likert scale, ranging from “extremely unhappy” (1) to “extremely happy” (5), and encompassed physical, social, and cognitive health domains (Supplement Perceived quality of life questionnaire). Total mean scores were computed across all items and for each of the three domains. To assess internal reliability of the modified version of the survey, Cronbach’s alpha was calculated using the *alpha* function in R’s *psych* package. Results revealed good internal consistency (α = 0.90).

### Covariates

Demographic covariates included age (years), income (≤ $30,000; $30,001– ≤ $55,000; ≥ $55,001), Hispanic ethnicity (yes or no), sex (male or female), and education (< high school/high school graduate, some college or vocational school, college graduate or graduate/professional school). Given that depression is increasingly recognized as an important CVD risk factor [29] and is known to adversely affect QoL [30], we included depression as a confounder in the final model to assess its association with CVH and PQoL. To determine the presence and severity of depressive symptoms, we used a validated, shortened form (10 items) [31] of the original 20-item Center for Epidemiologic Studies Depression Scale (CES-D-10) [32]. Mild-to-moderate depressive symptomology was defined as a CES-D-10 score of 10 or higher. Current depression status was defined as evidence of mild-to-moderate depressive symptomology and/or self-reported use of antidepressant. Participants with a score of 10 or greater were also mailed information about being at risk for depression and treatment options using an IRB approved letter.

### Statistical analysis

Participant characteristics were reported according to CVH categories from LS7 components and differences were tested with ANOVA for continuous variables and chi-square tests for categorical variables. To examine the association of overall CVH categories, continuous CVH score, and each LS7 component with PQoL, we conducted multivariable linear regression. We adjusted the models for age, Hispanic ethnicity, sex, income, education, and current history of depression. We also tested for effect modification of BMI and smoking with sex and BMI with Hispanic ethnicity by including an interaction term in the linear models (i.e., BMI*sex, BMI*Hispanic). All analyses were performed using SAS version 9.4 (SAS Institute, Cary, NC) and R software, version 4.4.0 (R Core Team, Vienna, Austria).

## Results

### Study population

Of the 602 participants who were enrolled in the study, 19 participants were excluded from the analysis due to missing data on at least one of the LS7 components, leaving a final analytic sample of 583 participants. Overall, the participants had a mean (SD) age of 58.7 (11.0) years, with 56% of the sample being female and 42% identifying as Hispanic. Approximately 30% of the participants reported experiencing mild or significant depressive symptoms and/or were taking antidepressants. The mean (SD) values for BMI, systolic BP (SBP), diastolic BP (DBP), and total cholesterol were 28.6 (5.9) kg/m^2^, 126.9 (17.4) mmHg, 72.9 (10.3) mmHg, and 188.7 (35.4) mg/dL, respectively. The median (IQR) for fasting glucose plasma and MVPA were 98.9 (92.1, 107.1) mg/dL and 21.3 (9.9, 38.8) minutes/day, respectively. The mean (SD) composite LS7 score was 8.3 (2.4). The median (IQR) for total score and the physical, cognitive, and social domains of PQoL were 4.0 (3.4, 4.4), 3.8 (3.0, 4.5), 4.0 (3.5, 4.5), and 4.0 (3.5, 4.5), respectively.

Participant demographic and clinical characteristics by CVH category are in Table 2. Being in the ideal CVH category was associated with younger age, female sex, higher education, and higher income, while Hispanics were less likely than non-Hispanics to have ideal CVH. Poor CVH was associated with a current history of depression. Lower PQoL score, both total score and in the physical domain, was associated with poorer CVH.

**Table 2:** Participant characteristics for the sample (n = 583), stratified by CVH category with significant differences between CVH groups tested with ANOVA (continuous variables) and chi-square tests (categorical variables)

Characteristic	Ideal^a^ (n = 189) N (%), Mean (SD), or Median (IQR)	Intermediate (n = 186) N (%), Mean (SD), or Median (IQR)	Poor (n = 208) N (%), Mean (SD), or Median (IQR)	*p*
Age (years)	55.7 (11.1)	58.5 (11.0)	61.7 (10.0)	< .0001
Female	123 (65.1)	94 (50.5)	107 (51.4)	0.006
Hispanic	63 (34.4)	71 (40.6)	98 (49.3)	0.028
*Education*				< .0001
High school or less	19 (10.1)	26 (14.0)	49 (23.6)	
College/vocational	37 (19.6)	59 (31.7)	67 (32.2)	
Higher education	131 (69.3)	99 (53.2)	92 (44.2)	
*Income*				< .0001
≤ $30,000	38 (20.1)	64 (34.4)	77 (37.0)	
$30,001– ≤ $55,000	37 (19.6)	44 (23.7)	52 (25.0)	
> $55,000	114 (60.3)	78 (41.9)	79 (38.0)	
Current history of depression^2^	40 (21.2)	59 (31.7)	73 (35.1)	0.008
Currently smoking	2 (1.1)	16 (8.6)	25 (12.0)	0.0001
BMI (kg/m^2^)	25.1 (4.6)	28.7 (5.6)	31.7 (5.5)	< .0001
*Blood Pressure (mmHg)*				
SBP	117.6 (13.8)	125.6 (15.6)	136.6 (16.9)	< .0001
DBP	69.3 (9.0)	72.8 (9.8)	76.2 (10.7)	< .0001
Fasting plasma glucose (mg/dL)	94.3 (88.6, 98.9)	98.5 (92.0, 105.1)	105.7 (99.0, 120.7)	< .0001
Total cholesterol (mg/dL)	184.4 (27.0)	189.3 (36.0)	191.9 (41.0)	0.105
MVPA (min/day)	35.7 (24.5, 50.4)	22.8 (11.5, 38.0)	10.1 (4.8, 19.1)	< .0001
*Healthy diet score*				< .0001
Poor	30 (15.9)	77 (41.4)	118 (56.7)	
Intermediate	120 (63.5)	93 (50.0)	84 (40.4)	
Ideal	39 (20.6)	16 (8.6)	6 (2.9)	
Total PQoL score, median (IQR)	4.1 (3.5, 4.5)	4.1 (3.4, 4.4)	3.8 (3.3, 4.2)	< .0001
*PQoL domains, median (IQR)*				
Physical	4.3 (3.5, 4.8)	4.0 (3.3, 4.5)	3.5 (2.8, 4.0)	< .0001
Cognitive	4.0 (3.5, 5.0)	4.0 (3.5, 5.0)	4.0 (3.5, 4.5)	0.809
Social	4.0 (3.5, 4.5)	4.0 (3.5, 4.7)	4.0 (3.3, 4.3)	0.086

^a^Poor defined as having a CVH score of 0-7; intermediate, 8-9; and ideal, 10- 14

^b^Defined as having a CES-D-10 score of 10 or higher, or self-reported antidepressant use

### Distribution of LS7 components

**Fig. 1:**
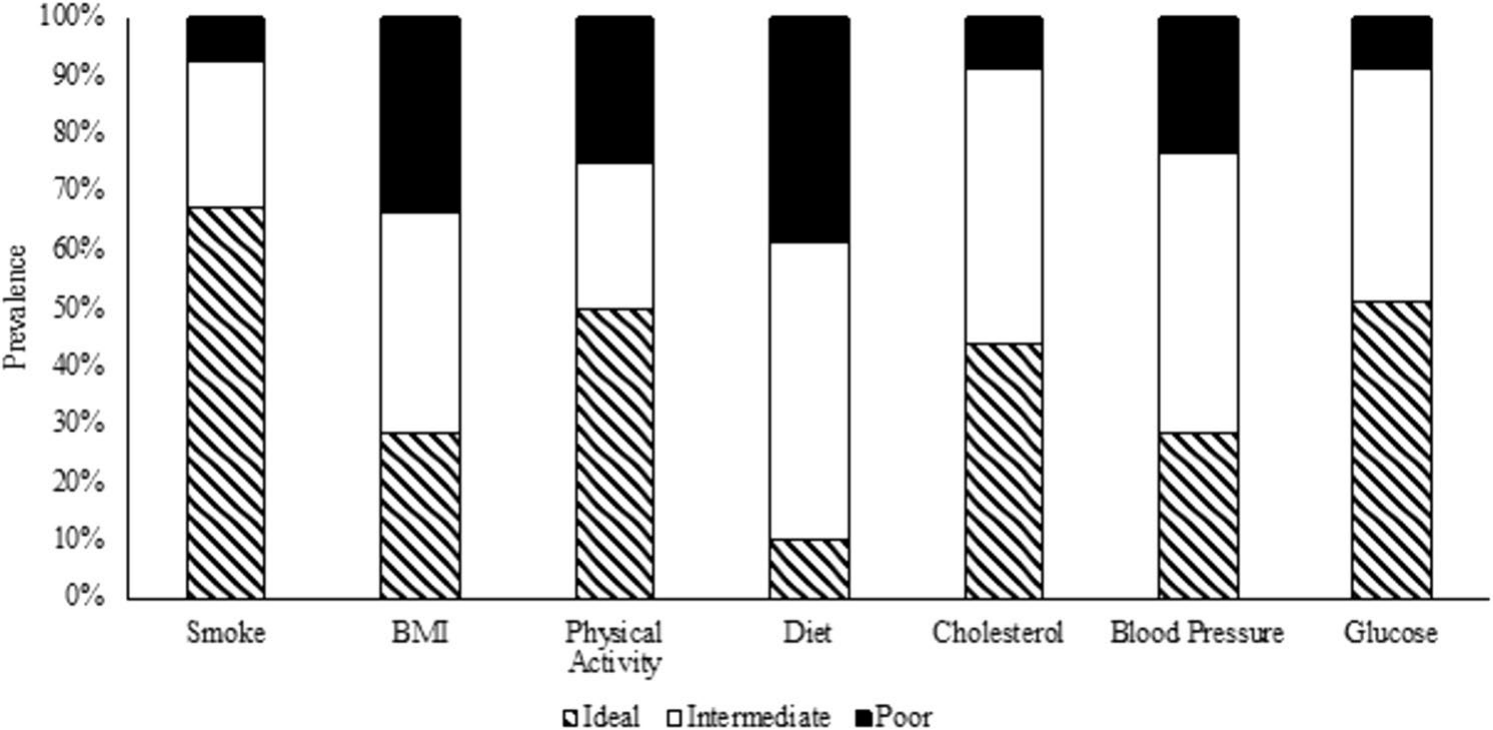
Proportion of participants meeting each LS7 component in ideal, intermediate, and poor CVH categories

Approximately 5% of the participants had 0 ideal components, and only two participants had all seven ideal components (Supplemental Figure 2). Figure 1 shows the proportion of LS7 components among the sample. The proportion of participants categorized as ideal was highest for the smoking (67%), followed by the physical activity (50%) components (Fig. 1). The proportion of participants meeting the ideal category was lowest for the dietary component (11%). Among the CVH health factors, ideal fasting plasma glucose concentration was the most prevalent (51%), whereas ideal BP was the least prevalent (29%) (Fig. 1). The prevalence of ideal total cholesterol was 44% and ideal BMI was 25% (Fig. 1).

### Adjusted associations between CVH and PQoL

Table 3 shows regression results examining the associations between the three CVH measures (CVH category, total CVH score, and number of ideal LS7 components) and total/domain specific PQoL. Overall, CVH was significantly associated with total PQoL and the physical domain of PQoL. Compared to those who had poor CVH, participants with ideal CVH had a higher mean total PQoL score [β (95% CI): 0.22 (0.08, 0.36)] and higher mean score on the physical domain [β (95% CI): 0.57 (0.40, 0.74)]. Similarly, participants with intermediate CVH had a higher mean total PQoL score [β (95% CI): 0.22 (0.09, 0.35)] and higher mean score on the physical domain [β (95% CI): 0.42 (0.26, 0.58)] compared to those with poor CVH. Results for total CVH score showed a similar association with total and physical domain PQoL. A one-point increase in CVH score was associated with a 0.04-point (95% CI = 0.02, 0.07) increase in total PQoL, and a 0.12-point (95% CI = 0.09, 0.15) increase in PQoL physical domain. Participants with more ideal LS7 components had higher total and physical PQoL scores. Full model results for CVH categories and PQoL total/domains are presented in Supplemental Table 1. Other full models are not shown; however, results were similar across models, and notably, there was an inverse association between depression and PQoL in all models.

**Table 3:** Multivariable linear regression analysis examining the association of CVH with total PQoL and PQoL domains (β (95% CI)) adjusted for age, sex, Hispanic ethnicity, income, education, and depression

Variable	Total PQoL	PQoL-Physical	PQoL-Cognitive	PQoL-Social
CVH category*				
Intermediate CVH Ideal CVH	0.22 (0.09, 0.35)	0.42 (0.26, 0.58)	0.08 (−0.09, 0.26)	0.13 (−0.01, 0.28)
	0.22 (0.08, 0.36)	0.57 (0.40, 0.74)	−0.03 (−0.21, 0.16)	0.06 (−0.09, 0.22)
Total CVH score	0.04 (0.02, 0.07)	0.12 (0.09, 0.15)	−0.01 (−0.04, 0.03)	0.01 (−0.02, 0.04)
Number of ideal components	0.05 (0.02, 0.09)	0.14 (0.09, 0.19)	0.002 (−0.05, 0.05)	0.02 (−0.03, 0.06)

*Reference value: poor CVH

**Fig. 2:**
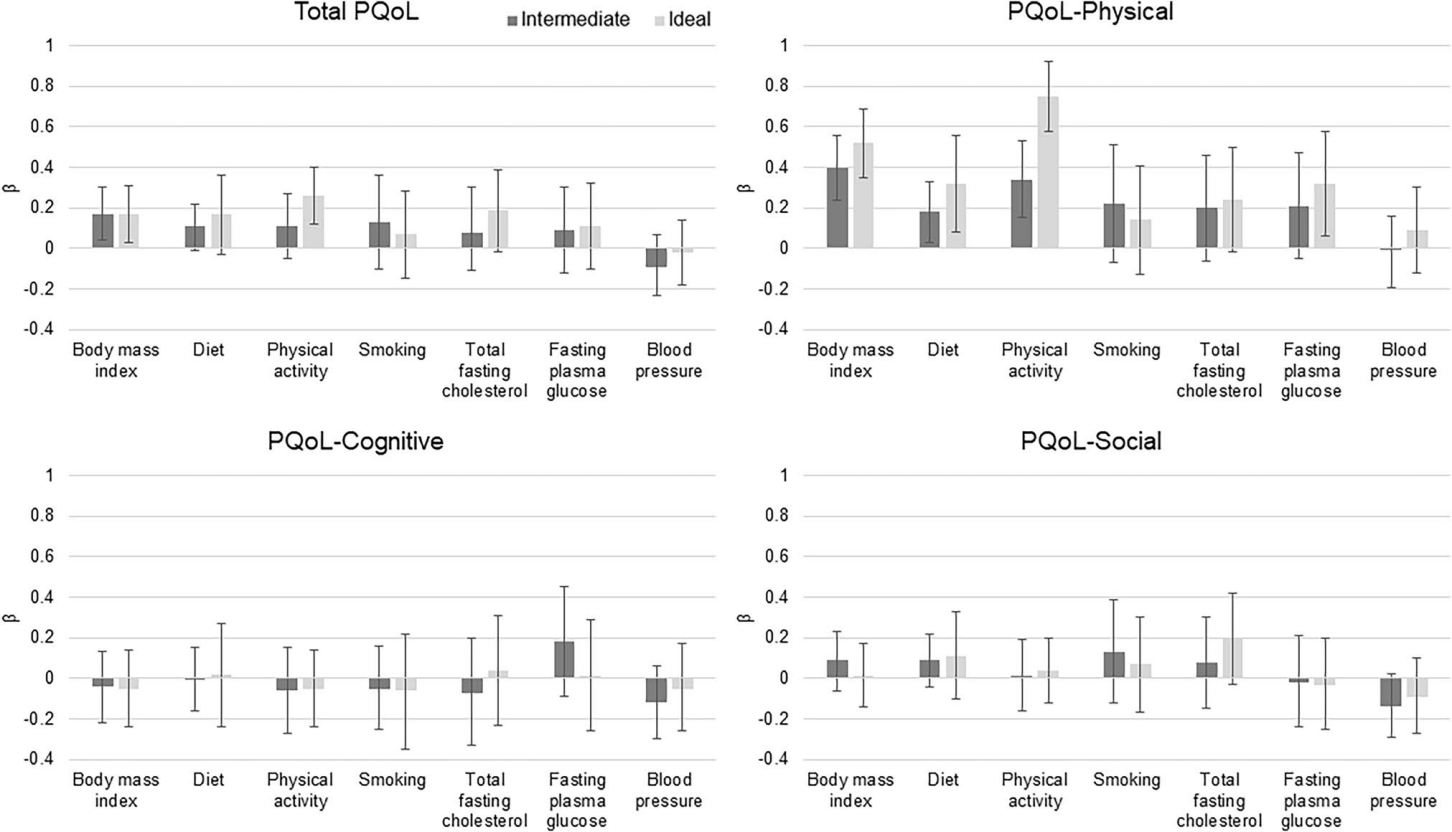
Multivariable linear regression results for individual LS7 components (modeled separately) and associations with total PQoL and domains (β (95% CI)). Reference category for each health behavior or factor is poor. Models were adjusted for age, Hispanic/Latino ethnicity, sex, income, education, and depression

After adjusting for covariates including depression, BMI and PA were significantly associated with total PQoL (Fig. 2). BMI, diet, and PA were significantly associated with the PQoL physical domain. Participants with ideal BMI had a higher mean PQoL score [β (95% CI): 0.17 (0.03, 0.31)] and higher mean score on the physical domain [β (95% CI): 0.52 (0.34, 0.69)] compared to those with a poor BMI. Similar results were found when comparing intermediate to poor BMI groups. Moving from intermediate to ideal groups, the diet and PA components showed increasing strength of associations with the physical domains of PQoL when compared to poor diet and PA. In examining the association between PA and total PQoL, the ideal group had a significantly higher PQoL compared to the poor group [β (95% CI): 0.26 (0.12, 0.40)], but results were not significant for the intermediate-poor group comparison. Fasting plasma glucose was the only health factor significantly associated with the physical PQoL domain; compared to the poor group, those with ideal FPG had higher mean scores [β (95% CI): 0.32 (0.06, 0.58)].

Significant BMI-by-sex interactions were observed for both total PQoL (p-interaction = 0.01) and the PQoL social domain (p-interaction = 0.01), as well as a smoking-by-sex interaction for the PQoL physical domain (p-interaction = 0.004). Table 4 presents analyses for BMI and smoking, stratified by sex. Women in the intermediate [β (95% CI): 0.35 (0.18, 0.53)] or ideal [β (95% CI): 0.17 (−0.01, 0.35)] BMI category, compared to the poor category, had higher total PQoL scores, with significant results only for the intermediate group. For men, there was no significant difference in total PQoL between intermediate and poor categories; however, those in the ideal category had higher total PQoL compared to the poor group [β (95% CI): 0.24 (0.01, 0.48)]. In both men and women, the ideal BMI group had a significantly higher PQoL scores in the physical domain compared to the poor group; however, for the intermediate-poor category comparison, this relationship was more strongly apparent in women. Also, women in the intermediate BMI category had higher PQoL scores on the social domain compared to those in the poor BMI category [β (95% CI): 0.31 (0.12, 0.51)]. For smoking, compared to the poor category, women in the intermediate [β (95% CI): 0.57 (0.14, 1.00)] category had higher mean PQoL scores in the physical domain. However, this relationship was not observed in men.

**Table 4:** Multivariable linear regression analysis examining the association of intermediate and ideal BMI and smoking with total PQoL scores and domains of PQoL, stratified by sex (β (95% CI))

	Females		Males			Females		Males		
	BMI*					Smoking**				
	Intermediate	Ideal	Intermediate	Ideal	P-Int	Intermediate	Ideal	Intermediate	Ideal	P-Int
Total PQoL	0.35 (0.18, 0.53)	0.17 (−0.01, 0.35)	0.01 (−0.18, 0.20)	0.24 (0.01, 0.48)	0.01	0.24 (−0.09, 0.57)	0.09 (−0.21, 0.40)	−0.02 (−0.35, 0.31)	0.02 (−0.30, 0.33)	0.23
PQoL-Physical	0.56 (0.34, 0.79)	0.55 (0.32, 0.77)	0.25 (0.02, 0.47)	0.52 (0.24, 0.79)	0.07	0.57 (0.14, 1.00)	0.32 (−0.08, 0.72)	−0.12 (−0.51, 0.27)	−0.02 (−0.39, 0.36)	0.04
PQoL-Cognitive	0.06 (−0.19, 0.30)	−0.13 (−0.37, 0.12)	−0.12 (−0.37, 0.12)	0.11 (−0.19, 0.41)	0.17	−0.06 (−0.51, 0.39)	−0.12 (−0.54, 0.29)	−0.05 (−0.46, 0.37)	−0.02 (−0.42, 0.38)	0.75
PQoL-Social	0.31 (0.12, 0.51)	0.02 (−0.17, 0.21)	−0.11 (−0.33, 0.10)	0.10 (−0.16, 0.37)	0.004	0.15 (−0.21, 0.50)	0.03 (−0.30, 0.36)	0.06 (−0.31, 0.42)	0.05 (−0.30, 0.40)	0.51

*Reference value is poor BMI category (BMI ≥ 30 kg/m^2^)

**Reference value is poor smoking category (current smoker)

There was also a significant BMI-by-Hispanic ethnicity interaction for the PQoL physical domain (p-interaction = 0.03). Table 5 presents analyses for BMI stratified by Hispanic ethnicity. Stratified results revealed significant associations of both ideal and intermediate BMI with PQoL scores in the physical domain for both Hispanics and non-Hispanics. However, the effect sizes were larger for non-Hispanics when compared to the Hispanics.

**Table 5:** Multivariable linear regression analysis examining the association of intermediate and ideal BMI with total PQoL scores and domains of PQoL, stratified by Hispanic/Latino ethnicity

BMI*	Total PQoL	PQoL-Physical	PQoL-Cognitive	PQoL-Social
*Hispanic/Latino*				
Intermediate Ideal	0.19 (−0.002, 0.38) 0.13 (−0.10, 0.36)	0.27 (0.04, 0.50) 0.33 (0.05, 0.61)	0.13 (−0.12, 0.38) −0.17 (−0.47, 0.13)	0.16 (−0.05, 0.36) 0.10 (−0.14, 0.36)
*Non-Hispanic/Latino*				
Intermediate Ideal	0.18 (0.003, 0.36) 0.20 (0.02, 0.38)	0.57 (0.36, 0.79) 0.69 (0.47, 0.92)	−0.17 (−0.40, 0.07) −0.02 (−0.26, 0.22)	0.03 (−0.17, 0.24) −0.06 (−0.27, 0.15)
P-Interaction	0.72	0.03	0.07	0.86

*Reference value is poor BMI category (BMI ≥ 30 kg/m^2^)

## Discussion

This study found overall CVH to be significantly associated with PQoL: participants with intermediate or ideal CVH categories had higher mean PQoL scores compared to participants in the poor CVH category; this was true for total PQoL and was largely driven by scores in the physical domain. Results were similar when examining total CVH score and number of ideal LS7 components; as total CVH score and number of ideal LS7 components increased, PQoL scores increased. Our results confirmed our initial hypothesis, with the caveat that in our study CVH does not appear to play a role in social and cognitive PQoL. When examining LS7 components separately, we found that compared to being in the poor category, being in the intermediate and/or ideal category for BMI, diet, PA, and FPG was associated with higher PQoL scores, particularly in the physical domain.

Our study supports a growing body of evidence that PQoL relates to objective and self-reported measures of CVH. Agostinis-Sobrinho et al. found a positive association between ideal CVH and health-related QoL (HRQoL) in Portuguese adolescents [33]. Similar associations were found in the NHANES and BRFSS data, which indicated that both ideal and intermediate CVH were linked to better overall health status and fewer physically and mentally unhealthy days in the past month [13, 14]. A study by Bergman et al. also found a favorable CVH profile to be associated with better PQoL among a sample of Finnish employees [34]. This growing body of evidence suggests that overall CVH status may provide insights into an individual’s perceived quality of life across different domains; in turn, a positive perceived quality of life may indicate an individual’s desire or motivation to change their behaviors, which can potentially improve future health outcomes.

While the combined LS7 gives a general understanding of CVH, analyzing the individual components that constitute LS7 and their associations with PQoL may provide insights into specific behaviors or health outcomes that could be prioritized for targeted interventions. Bergman et al. reported higher mean PQoL scores for favorable levels of health behaviors and HbA1c, but not for BP or total cholesterol [34]. Similarly, our study found comparable associations between health behaviors (diet and PA), BMI and PQoL in both overall and physical domain, as well as fasting plasma glucose for the physical domain. Given that the PQoL scale reflects an individual’s happiness with different domains of their life, and happiness reflects their current mood [35], it is plausible that engaging in healthy behaviors is more immediately linked to how an individual perceives their life. Furthermore, it appears that this link is more likely to be perceived in the physical domain of PQoL, whereas the link between CVH and perceived cognitive or social domains is less apparent. Some of the null associations in the cognitive and social domains may be explained by no direct link with CVH components, such as diet is not likely to be associated with perceived social quality of life. Other null associations may be explained by the mismatch between participant perceptions of their quality of life and researcher assessed quality of life. For example, while increased physical activity is associated with better cognitive functioning assessed by a researcher [36], there is poor agreement between participant perception of their own cognitive functioning and assessed cognitive functioning [37].

We found sex to be a significant effect modifier in the association between BMI and PQoL. The sex differences were found to be significant only for women, particularly in relation to total PQoL and the social PQoL domain, specifically for those in the intermediate BMI category (overweight, BMI 25.0–29.9 kg/m^2^) as compared to the poor (obese) category. Prior research has suggested that social connection is more strongly associated with obesity in women than in men, especially in older women [38]. Furthermore, we observed that sex acted as a significant effect modifier in the association between smoking and the PQoL physical domain in women. Specifically, former women smokers had higher PQoL scores than current smokers. No significant differences were observed between never smokers and current smokers, possibly due to the limited number of active smokers in the study.

Although we found Hispanic ethnicity to be a significant modifier in the association between intermediate/ideal BMI and the physical PQoL domain, our examination of Hispanics and non-Hispanics separately revealed that the association of intermediate/ideal BMI with higher physical PQoL scores was apparent in both groups. However, the strength of the relationship was greater in non-Hispanics than Hispanics. This suggests that for non-Hispanics, weight status may be more closely linked to their self-rated physical health. It is possible that ethnic differences exist in terms of the most important factors contributing to PQoL. At the same time a growing body of evidence is recognizing that cardiovascular risk factor cut-offs may need to be adjusted (increased) for Hispanics as their biomarkers remain in healthy ranges at higher abdominal obesity [39].

While this study was cross-sectional, it does suggest that differences between sexes exist, and for women, perceived quality of life may provide insight into improving and maintaining healthy behaviors. This suggests that interventions for CVH may be tailored to address gender-specific barriers to healthy behaviors and target PQoL improvement. For example, prioritizing weight management in women may do more to improve PQoL than other factors, compared to men. Finally, interventions may need to be tailored differently for different ethnic groups to consider cultural norms and variations in perceived quality of life, compared to the general population.

There are some limitations to our study. First, since this is a cross-sectional study, there were inherent temporality issues (PQoL was measured at the same times as CVH) that need to be considered in the interpretation of the findings. Although the PQoL scale used in this study captures happiness with physical, cognitive, and social domains of life, it does not comprehensively assess QoL. Future studies should consider including a more comprehensive assessment of QoL, such as domains that cover sexual functioning, religious or spiritual well-being, and resilience in addition to the commonly assessed domains of social functioning, physical functioning, general health perceptions, and disease-specific symptoms [40]. Additionally, while we adjusted for common covariates in models of CVH and PQoL, there are likely some unmeasured confounders in this relationship, such as medication use or chronic illness which may bias the reported association sizes from their true values. Inclusion of a more comprehensive set of confounders would partially address this bias, however, residual confounding would still exist and bias associations [41]. Additionally, residual confounding may create spurious or mask associations in the interaction analyses reported (by sex and Hispanic ethnicity) [42]. Finally, the minor modifications of the LS7 and PQoL instruments were necessary to adapt to the protocol of this study. While the validity of these adaptations was not able to be assessed for this study sample, we believe that the validity is comparable to the original instruments.

In conclusion, better CVH is associated with better PQoL, driven by satisfaction with physical functioning. Interventions could account for sex and ethnicity. Environmental, sociocultural, and psychosocial factors should be examined in relation to potential racial/ethnic differences in LS7 components and PQoL.

## Supplementary Information

Below is the link to the electronic supplementary material.

Supplementary file1 (DOCX 294 KB)
