# Supplementary material for: Association between cardiovascular health and perceived quality of life in ethnically diverse adults: insights from the Community of Mine study using the American Heart Association’s Life’s Simple 7
Source: Qual Life Res. 2024 Dec 18;34(3):789–99. doi: 10.1007/s11136-024-03853-3 (PMC11920301; doi:10.1007/s11136-024-03853-3)
Supplement: Supplementary file 1 — Supplementary file1 (DOCX 294 KB) [file 11136_2024_3853_MOESM1_ESM.docx]

**Online Supplement**

**Supplement Figure 1.** Study sample enrollment flowchart.


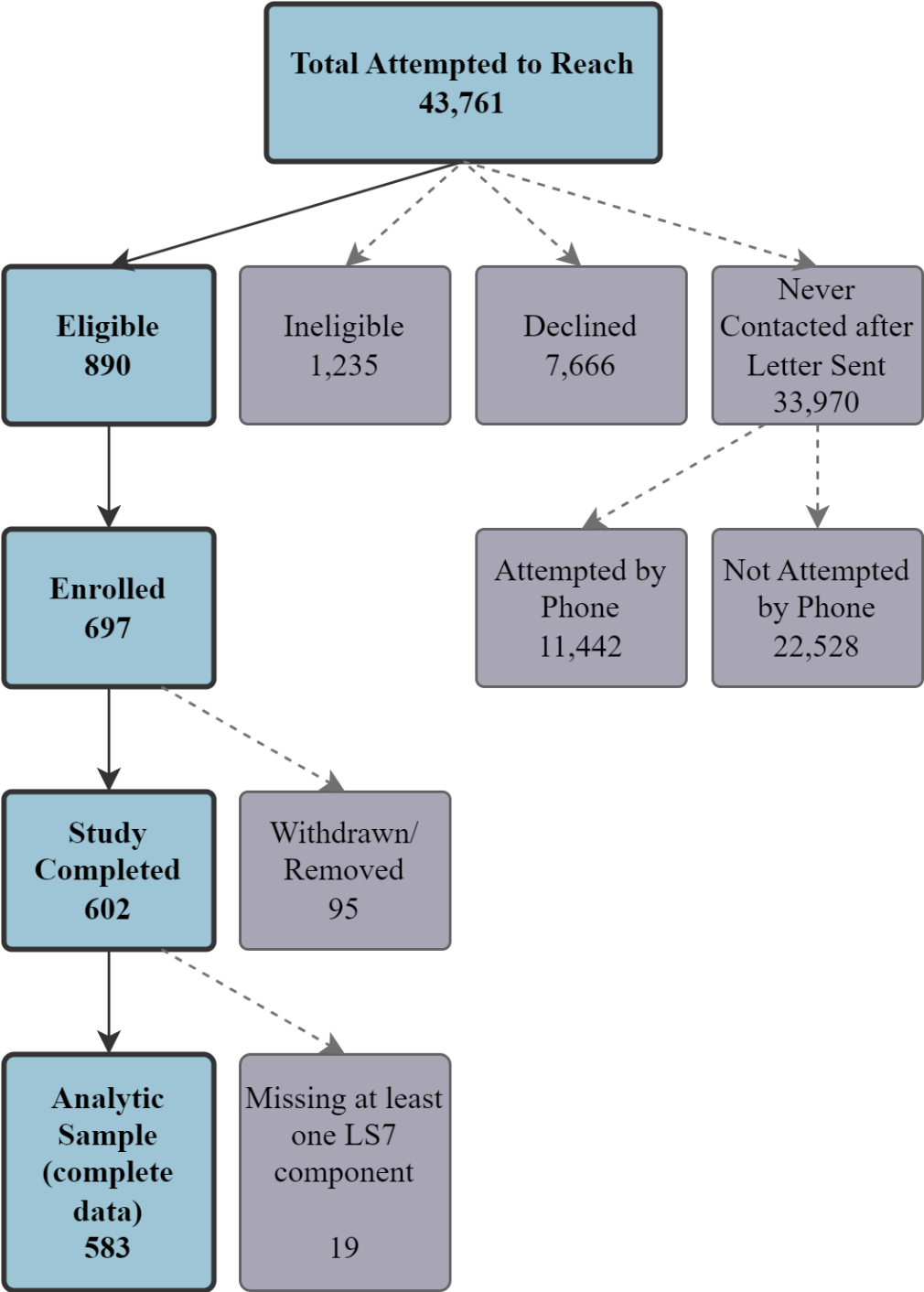


**Supplemental Figure 2**. Number of ideal LS7 components in the 583 participants with complete LS7 data.


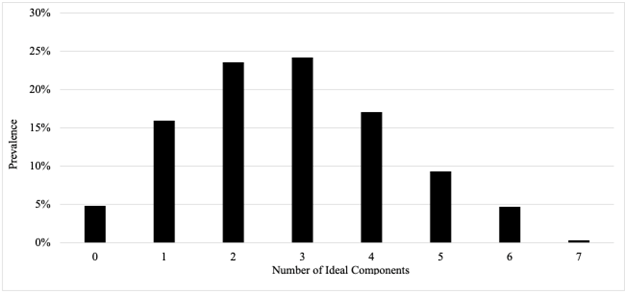


**Supplemental Table 1**. Full model summary of multivariable linear regression analysis examining the association of CVH categories with total PQoL and PQoL domains (β (95% CI)).

| Variable | PQoL Score | PQoL-Physical | PQoL-Cognitive | PQoL-Social |
| --- | --- | --- | --- | --- |
| CVH category  Intermediate CVH  Ideal CVH | 0.22 (0.09, 0.35)  0.22 (0.08, 0.36) | 0.42 (0.26, 0.58)  0.57 (0.40, 0.74) | 0.08 (-0.09, 0.26)  -0.03 (-0.21, 0.16) | 0.13 (-0.01, 0.28)  0.06 (-0.09, 0.22) |
| Age (per 5-year change) | 0.06 (0.04, 0.09) | 0.08 (0.05, 0.12) | 0.04 (0.001, 0.07) | 0.06 (0.03, 0.09) |
| Hispanic/Latino ethnicity | 0.16 (0.03, 0.28) | 0.10 (-0.05, 0.26) | 0.05 (-0.12, 0.22) | 0.23 (0.09, 0.37) |
| Female | 0.16 (0.05, 0.28) | 0.01 (-0.13, 0.15) | 0.04 (-0.11, 0.19) | 0.31 (0.18, 0.43) |
| Income  ≤$30,000    $30,001-≤$55,000 | -0.14 (-0.29, 0.004)  -0.20 (-0.34, -0.05) | -0.07 (-0.25, 0.11)  -0.19 (-0.37, -0.02) | -0.07 (-0.26, 0.12)  -0.06 (-0.25, 0.14) | -0.21 (-0.37, -0.05)  -0.24 (-0.40, -0.08) |
| Education  High school grad or less  Some college or vocational school | 0.04 (-0.14, 0.23)  -0.03 (-0.16, 0.11) | 0.16 (-0.07, 0.38)  0.03 (-0.13, 0.20) | 0.01 (-0.24, 0.25)  0.03 (-0.14, 0.21) | -0.02 (-0.22, 0.19)  -0.09 (-0.24, 0.06) |
| Current history of depression | -0.62 (-0.74, -0.49) | -0.68 (-0.83, -0.53) | -0.59 (-0.76, -0.43) | -0.58 (-0.72, -0.45) |

Reference groups: Poor CVH; income >$55,000; college graduate or graduate school

**Perceived quality of life questionnaire**

**I. QUALITY OF LIFE**

Please answer the questions by indicating how **happy** you are with the following.

| 1. How **happy** are you with… | Extremely unhappy | Somewhat unhappy | Neutral | Somewhat happy | Extremely happy |
| --- | --- | --- | --- | --- | --- |
| 1. your physical health (the health of your body) | _1_ | _2_ | _3_ | _4_ | _5_ |
| 1. how well you care for yourself, for example preparing meals, bathing, or shopping | _1_ | _2_ | _3_ | _4_ | _5_ |
| 1. how well you think and remember | _1_ | _2_ | _3_ | _4_ | _5_ |
| 1. the amount of walking you do | _1_ | _2_ | _3_ | _4_ | _5_ |
| 1. how often you get outside | _1_ | _2_ | _3_ | _4_ | _5_ |
| 1. how well you carry on a conversation, for example speaking clearly, hearing others, or being understood | _1_ | _2_ | _3_ | _4_ | _5_ |
| 1. how often you see or talk to your family and friends | _1_ | _2_ | _3_ | _4_ | _5_ |
| 1. the help you give to your family and friends | _1_ | _2_ | _3_ | _4_ | _5_ |
| 1. your contribution to your community | _1_ | _2_ | _3_ | _4_ | _5_ |
| 1. the kind and amount of recreation or leisure you have | _1_ | _2_ | _3_ | _4_ | _5_ |
| 1. how respected you are by others | _1_ | _2_ | _3_ | _4_ | _5_ |
| 1. the meaning and purpose of your life | _1_ | _2_ | _3_ | _4_ | _5_ |
|  | Extremely unhappy | Somewhat unhappy | Neutral | Somewhat happy | Extremely happy |
